# Zinc Amido-Oxazolinate Catalyzed Ring Opening Copolymerization and Terpolymerization of Maleic Anhydride and Epoxides

**DOI:** 10.3390/molecules25184044

**Published:** 2020-09-04

**Authors:** Muneer Shaik, Vamshi K. Chidara, Srinivas Abbina, Guodong Du

**Affiliations:** Department of Chemistry, University of North Dakota, 151 Cornell Street Stop 9024, Grand Forks, ND 58202, USA; muneer.shaik@und.edu (M.S.); vamshi700@gmail.com (V.K.C.); srini.abbina@gmail.com (S.A.)

**Keywords:** polyester, zinc catalysis, ring opening copolymerization, terpolymerization, maleic anhydride

## Abstract

Ring opening copolymerization (ROCOP) of epoxides and cyclic anhydrides has become an attractive approach for the synthesis of biodegradable polyesters with various compositions. Encouraged by the efficiency and versatility of a series of amido-oxazolinate zinc complexes, in this study they were shown to be active catalysts for the synthesis of unsaturated polyesters via ROCOP of maleic anhydride and various epoxides. The relative activity of epoxides in these reactions was observed to be styrene oxide > cyclohexene oxide > phenyl glycidyl ether, which could be correlated with the electronic and steric features of the substrate. To provide more structural possibilities for the polyesters, the difference in epoxide reactivity was exploited in an attempt to prepare block terpolymers from one anhydride and two epoxides. Terpolymerization was carried out in one or two steps in a single pot. The thermal characterization by thermogravimetric analysis (TGA) and differential scanning calorimetry (DSC) techniques suggested that the resulting materials were mostly random terpolymers.

## 1. Introduction

Polyesters are an important class of biodegradable and biocompatible polymers that possess various applications in drug delivery, orthopedic implants, artificial tissues, and commodity materials [1,2,3,4]. Typical synthetic methods leading to polyesters involve step growth polycondensation or ring-opening polymerization (ROP) [5,6]. The step-growth polymerization of diols and diacids/diesters usually requires high temperatures and long reaction times, and can lead to side reactions and low molecular weight polymers [7,8,9,10]. ROP of cyclic esters effectively produces polyesters with controlled structure and molecular weight, but the availability of cyclic monomers may be limited. Catalytic ring-opening copolymerization (ROCOP) of epoxides and cyclic anhydrides is another technique to produce polyesters that has gained increased attention in recent years [11,12]. This approach could possibly circumvent the limitations posed by the aforementioned methods while taking advantage of both. Specifically, a wide selection of diverse epoxides and cyclic anhydrides offers the structural flexibility and tunability of the step growth polymerization. The milder reaction conditions and potential controllability of the chain growth polymerization may lead to more economical production and well-defined polymer structures [13]. Additionally, ROCOP with two or more different epoxides or cyclic anhydrides provides endless possibilities in modifying the polymer backbone structure and thermomechanical properties. The increasing availability of bio-derived epoxides and cyclic anhydrides further enhances the attractiveness of the approach [14,15,16].

Generally, ROCOP of epoxides and cyclic anhydrides is believed to proceed through alternating insertion of the two monomers into the growing chain of alkoxide and carboxylate, a mechanistic scenario very similar to ROCOP of epoxides and CO_2_ that generates polycarbonates [17]. While the polycarbonate synthesis via ROCOP has been well-studied and various catalysts have been reported [18,19], ROCOP of epoxides and cyclic anhydrides for polyesters has seen a rapid growth only in the last decade, notably since the discovery made by Coates and co-workers that zinc β-diketiminate catalyzed ROCOP afforded polyesters with high molecular weight and narrow dispersity [20]. Most of the catalysts are based on Lewis acidic metals supported by various ligands, such as Cr [21,22,23,24], Co [25,26,27,28], Zn [29,30,31,32,33], Al [34,35], and Mg [36,37]. Metal-free organocatalysts have been developed more recently [38,39,40,41]. These progresses have led to highly active and selective catalysts, well-controlled polymer structures and molecular weights, and adjustable polymer sequences [42,43].

**Scheme 1:**
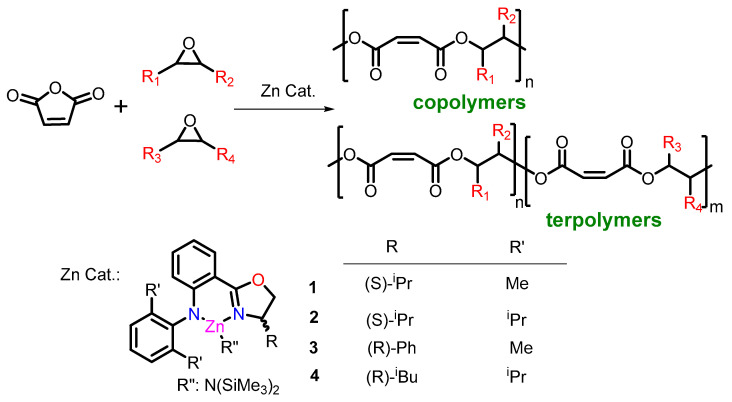
Ring opening copolymerization (ROCOP) of anhydrides and epoxides catalyzed by amido-oxazolinate zinc complexes.

A series of chiral amido oxazolinato zinc complexes, analogous to the widely studied β-diketiminato zinc catalysts, have been shown to be effective in the synthesis of biodegradable polymers including polycarbonates [44], polylactides [45], and polyesters [46] via catalytic ring opening polymerization and copolymerization. The zinc based catalysts have been attractive because zinc is relatively abundant, non-toxic, and biocompatible, suitable for applications in biomedical fields [47]. The introduction of the stereogenic centers in these catalysts has led to stereocontrol of the resultant polymers [44,45], which may influence their thermal and physical properties and their utility. Encouraged by the high efficiency of the amido oxazolinato zinc catalysts in ROCOP, with turnover frequency up to 4000 h^−1^ [46], we are interested in expanding the substrate scope of these zinc catalysts in ROCOP with an eye on the possible stereocontrol, and utilizing the catalytic versatility to generate polyesters with structural diversity by terpolymerization. Herein, we described our efforts in these directions (Scheme 1) and investigated the thermomechanical properties of the terpolymers. The focus of the anhydride substrate here is maleic anhydride (MA), because MA can be derived from biomass [48] and the ROCOP with MA leads to formation of unsaturated polyesters, which have been widely used in a variety of applications based on the post-polymerization modification of C=C double bonds [49,50].

## 2. Results and Discussions

### 2.1. Optimizing Reactions with MA/CHO

Previously we have investigated the zinc (complexes **1**–**4**) catalyzed ROCOP using styrene oxide (SO) as a representative electron withdrawing epoxide for preparation of semi-aromatic polyesters with different cyclic anhydrides [31]. Among them, maleic anhydride (MA) showed the highest selectivity towards polyester formation. Hence, we focused on MA and cyclohexene oxide (CHO), an aliphatic epoxide, to expand the substrate scope of the catalysis. Because CHO could easily undergo homopolymerization at elevated temperatures in the presence of a catalyst, the initial reaction conditions were varied in an attempt to minimize the formation of ether linkages in the ROCOP of CHO and MA. Following the standard conditions identified earlier with SO, the reaction was carried out with CHO:MA:**1** ratio of 100:100:1 in toluene at 100 °C. Complete conversion of CHO was achieved in 16 h with 53% poly(ester) linkage with *M*_n_ of 5.9 kg/mol and dispersity (Đ) of 1.29. Despite of the moderate molecular weight, 47% poly(ether) was formed under this condition (Table 1, entry 2). To lower the concentration of CHO, a solution of CHO in toluene was slowly added to a solution of MA and cat-**1** in toluene over a period of 30 min (entry 3). The reaction reached 100% conversion of CHO in 24 h and afforded polymer with 73% poly(ester) linkages and lower molecular weight (*M*_n_ 2.7 kg/mol), presumably due to the dilution of reaction mixture. Using CHO:MA:cat**-1** ratio of 200:200:1, reaction afforded polymer with 61% ester linkages in 9 h with *M*_n_ of 2.3 kg/mol and Đ of 1.20 (entry 4). To see the effect of the dilution, the same reaction was repeated with 10 mL toluene instead of the previously used 2 mL. As expected, reaction required more time and *M*_n_ was decreased to 1.2 kg/mol without much change in the selectivity towards poly(ester) (entry 5). Under bulk conditions without solvent, homopolymerization of CHO was predominant, leading to 76% poly(ether) content (entry 6). The conversion reached a plateau due to the viscous nature of the reaction mixture that limited the reactivity between the two co-monomers in the reaction. Using excess MA in the reaction afforded low molecular weight polymers (entry 7). The trace amount of maleic acid and other protic impurities present in MA even after multiple sublimations may have acted as chain transfer agents that led to low molecular weight polymers. The absence of the amide N(SiMe_3_)_2_ end group in the polymer indicated that it is not the initiating group for ROP. So it is likely that maleic acid served as the initiating group in the reaction. The similar result was observed in the last run, where excess co-monomers resulted in low molecular weight polymers (entry 8). Without catalyst, the reaction of CHO and MA afforded no poly(ester) or poly(ether) at 100 °C and extended reaction time. (entry 1). Considering both reactivity and selectivity, an epoxide:anhydride:catalyst ratio of 200:200:1 at 100 °C was employed in the later runs.

### 2.2. Comparison of Epoxides in ROCOP with MA

**Figure 1:**
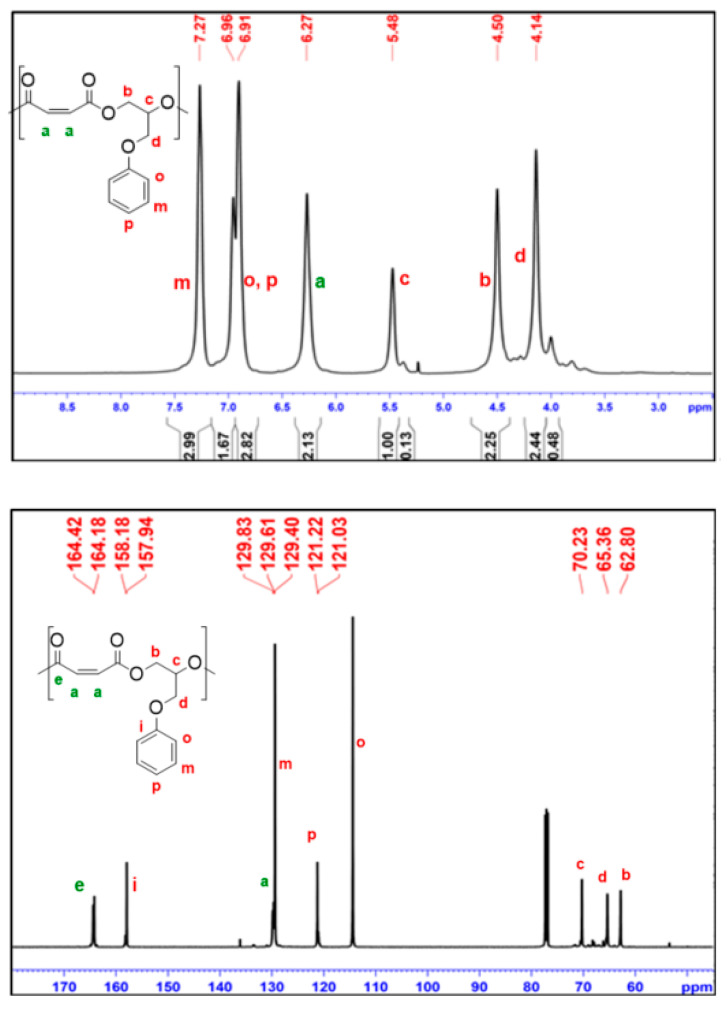
^1^H (top) and ^13^C NMR (bottom) spectra of the polyester obtained from the reaction between phenyl glycidyl ether (PGE) and MA in the presence of cat-**1**.

Given the difference observed between CHO and SO, we were interested in further comparing the reactivity of different epoxides by including a third epoxide, phenyl glycidyl ether (PGE) in the copolymerization with MA catalyzed by cat-**1**. The results are summarized in Table 2. Clearly, the reactivity of PGE in ROCOP with MA was much lower compared to SO (entry 1) and CHO (entry 2), requiring longer time (48 h) for a complete conversion of PGE (entry 3). Conversely, the selectivity towards ester linkages was high, reaching up to 95% under the employed reaction conditions. The molecular weight was also higher with a broad dispersity. The ^1^H and ^13^C NMR of the purified polyester are shown in Figure 1. The olefinic proton peaks are observed at 6.27 ppm, and the ester bridge signals are observed at 5.48 (methine) and 4.50 (methylene) ppm. The assignments are confirmed by 2D NMR techniques (see Figures S5 and S6 in the Supplementary Material). These results showed minimal amount of ether signals from the homopolymerization of PGE and are in support of the alternating nature of the polyester structure. In none of the cases isomerization or crosslinking of the *cis*-double bond in the polymer was noticed.

**Scheme 2:**
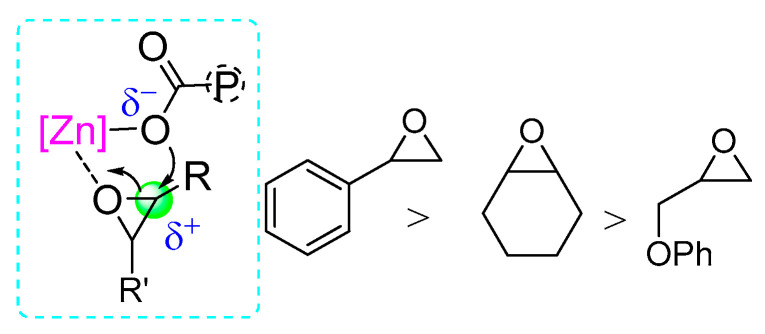
Chain propagation step explaining the reactivity of epoxides.

The observed relative reactivity of epoxides, SO > CHO > PGE, can be explained approximately based on their electronic factors. Generally, zinc-catalyzed ROCOP involves the nucleophilic attack of chain ends on epoxide and anhydride monomers alternatingly in the chain propagation. The ring opening reaction at the epoxide is electronically controlled, i.e., the attack at the electron-deficient site is favored (Scheme 2). SO features an electron withdrawing phenyl substituent that facilitates the nucleophilic attack of propagating carboxylates on the epoxide ring, leading to a comparably fast reaction. In comparison, the electron donating nature of substituents in PGE and CHO renders them less active for the epoxide ring-opening event. The electronic dependence on the epoxide monomer resembles the effect of catalysts and selectivity in ROCOP of SO and MA [31]. The fact that both SO with an electron withdrawing group and PGE with an electron donating group give higher selectivity toward ester linkages suggests that the propensity of CHO toward homopolymerization (to polyether) is mostly due to the ring strains in CHO.

### 2.3. Effect of Catalysts and Anhydrides in ROCOP

With CHO as a representative aliphatic epoxide, we further investigated the effect of catalysts bearing various substituents on the zinc-catalyzed ROCOP. As seen in Table 3 and the NMR spectra in Figures S1–S4, complexes **1**–**4** showed comparable activity in ROCOP of MA and CHO, irrespective of their steric and electronic features, resulting in complete conversion of CHO in 2–4 h and comparable molecular weight of polymers (entries 1–4). However, the selectivity towards ester linkages was decreased (37–44% polyester). In these cases, MA was not completely consumed in the reaction. The observed low selectivity towards ester linkages (vs. ether linakge) of these zinc catalysts are not uncommon, but nearly exclusive ester linkage formation has been achieved with other zinc catalysts [20]. We further explored the effect of cyclic anhydride structure on ROCOP. High conversion of CHO was observed within 3-6 h with phthalic anhydride (PA) (entries 5–8), though the selectivity towards polyester and the molecular weights were low (34–43% polyester). ROCOP with succinic anhydride (SA) (entries 9–12) generally afforded polymers with higher selectivity (50–66% polyester) and higher molecular weights, but required longer reaction times (6–11 h). Cat-**2** bearing isopropyl substituent on the anilinido moiety as well as on the oxazoline ring afforded 74% selectivity toward ester and *M*_n_ of 4.2 kg/mol, however, 100% conversion was not achieved in this case (entry 10). Overall, the relative activity of the three cyclic anhydrides in the reaction could be attributed to the ring strain in the cyclic anhydrides: PA ≈ MA > SA, while the structural features of catalysts **1–4** seemed to exert no clear trend in their reactivity. This latter observation is in contrast with the ROCOP of SO and cyclic anhydrides, where both electronic and steric features of catalysts play a noticeable role [31].

### 2.4. Terpolymerization of MA and Two Epoxides

One common strategy for tuning the polymer properties such as glass transition temperature (*T*_g_) is to incorporate additional monomers to form block or random copolymers [51,52,53,54]. Given the versatility of the current zinc catalysts for various epoxides and anhydrides, we were interested to see if terpolymerization could be achieved and how it could affect the properties of the resulting terpolymers. For this purpose we selected the three epoxides (SO, CHO, and PGE) differing in their rates of reactions with maleic anhydride (MA) to synthesize terpolymers from MA and two epoxides catalyzed by cat-**1**. Selected results are summarized in Table 4.

**Figure 2:**
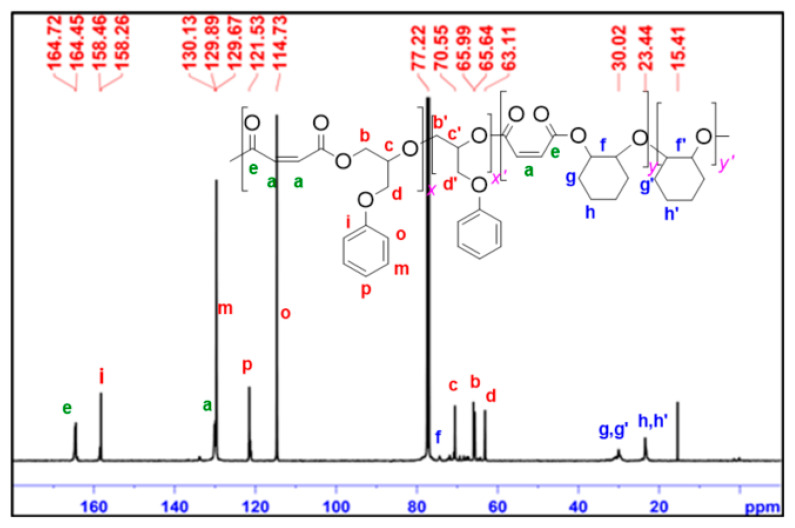
^13^C NMR spectrum of terpolymer poly(CHO-PGE-MA) (Table 4, entry 2).

To take advantage of the reactivity difference of epoxides, we first carried out the terpolymerization of two equivalents of MA with one equivalent of CHO and one equivalent of PGE in one pot, in an attempt to obtain diblock polyesters in a single step [55,56,57]. Monitoring the reaction by ^1^H NMR spectroscopy showed that SO was consumed within a few hours while the complete conversion of the second epoxide PGE was much slower, as expected from the activity difference of SO and PGE. However, the molecular weight of the resulting polymer was only 4.3 kg/mol, much less than the theoretical value estimated from the catalyst loading and conversion (Table 4, entry 4). Running the reaction in one pot but two steps by sequential addition of CHO and PGE gave similar results; particularly, the molecular weights obtained after the complete consumption of first addition of CHO showed little increase during the second step when PGE was added (Table 4, entry 1). Reversing the order of the two steps, i.e., adding PGE first followed by CHO, led to somewhat higher molecular weight but still lower than the theoretical value (Table 4, entry 2). A representative ^13^C NMR spectrum of the terpolymer p(CHO-PGE-MA) is shown in Figure 2 and the ^1^H NMR spectrum, as well as the spectra for other terpolymers, are shown in the SM (Figures S7–S18). The ratio of the PGE-derived ester to the CHO-derived ester is about 2.7:1. The relatively low CHO component as ester may be a result of CHO homopolymerization. Different combinations of two epoxides afforded similar results in terms of the molecular weights and compositions (entries 5 and 6). Thus it appeared that the diblock polyesters were not obtained under these conditions. Instead, they were mostly likely random polymers. We ascribed this to the presence of chain transfer agents and to a transesterification reaction that led to the scrambling of the ester linkages and shorter chains.

### 2.5. Thermal Properties of Copolymers and Terpolymers

To explore the effect of the terpolymer structure, their thermal properties were next studied with differential scanning calorimetry (DSC) and thermogravimetric analysis (TGA) techniques. For comparison, we first investigated the behaviors of copolyesters, p(CHO-MA), p(SO-MA) and p(PGE-MA) synthesized in the present study, the results of which are summarized in Table 5. As expected, the glass-transition temperatures (*T_g_*) of the polymers are dependent on the structure of the epoxide monomers. The polymer p(CHO-MA) has the highest *T*_g_ at 63 °C among the three, which can be attributed to the presence of rigid cyclohexyl ring structure in the polymer main chain. P(SO-MA) has a *T*_g_ of 39 °C, and p(PGE-MA) has a lowest *T*_g_ of 30 °C, which can be rationalized on the basis of the steric bulk of the substituents. For the terpolymer p(CHO-PGE-MA), a single glass transition temperature was observed at 41–44 °C, which falls between the *T_g_* of p(CHO-MA) and p(PGE-MA). The other terpolymers also displayed a single glass transition that mostly fell between the corresponding copolymers. These results suggested that the terpolymers were mostly random polymers, in agreement with the assignments from NMR and gel permeation chromatography (GPC) studies earlier.

**Figure 3:**
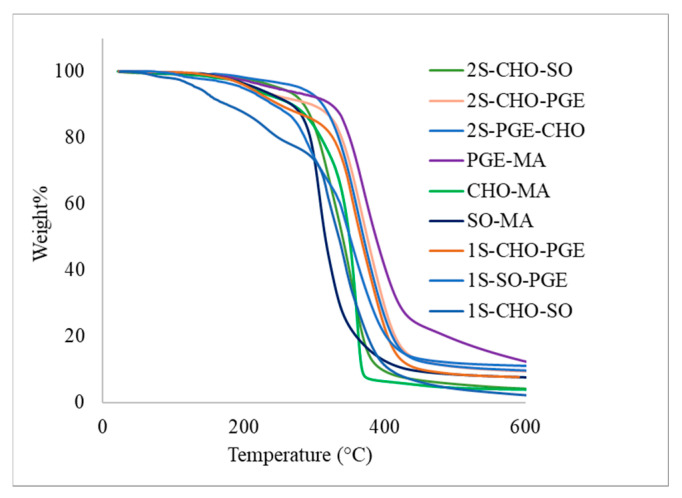
TGA profiles of the copolymers and terpolymers (1S for one-step and 2S for two-step reactions).

The thermal stability of these polyesters were examined by TGA, and the results are summarized in Figure 3 and Table 5. Most of the polymers have thermal degradation temperature above 200 °C, typical of polyester behavior. Generally, the terpolymers showed a single stage thermal decomposition profile and the *T*_−50%_ values of the terpolymers fall in between the *T*_−50%_ of the corresponding copolymers. The significant amount of residues may be attributed to the crosslinking of the C=C double bonds in the polymer chain. It was also noted that a poorly resolved second peak could be observed in the differential thermal analysis (DTA) plot (Figure S19) for some cases. The observations suggested that the resulting polymers are likely random terpolymers, but perhaps with enriched segments of copolyesters.

## 3. Materials and Methods

Solvents used in polymerization reaction and ligand syntheses including toluene and hexanes were distilled under nitrogen over Na/benzophenone. Deuterated solvents purchased from Cambridge Isotope Laboratories (Tewksbury, MA, USA) and distilled over CaH_2_ and degassed prior to use and stored in a glovebox. HPLC grade THF was purchased from Fisher Scientific (Hampton, NH, USA) and used as received. Cyclohexene oxide, styrene oxide, and phenyl glycidyl ether were stirred over ground CaH_2_ overnight, vacuum transferred to a dry Schlenk flask, degassed by three freeze-pump-thaw cycles, and stored in a glovebox with 4 Å molecular sieves. Maleic anhydride, phthalic anhydride, and succinic anhydride were purified by sublimation under nitrogen. The zinc catalysts were prepared according to the literature procedures [58,59].

All reactions with air- and/or moisture sensitive compounds were carried out under dry nitrogen atmosphere using standard glovebox and/or Schlenk line techniques. 1D and 2D NMR experiments were recorded with a Bruker AVANCE-500 NMR spectrometer (Billerica, MA, USA) and the spectra were referenced to the residual peaks in CDCl3 (^1^H, and ^13^C). The microstructures of polyesters samples were characterized by ^13^C NMR spectra recorded at room temperature in CDCl_3_ with concentrations in the range 1–1.5 mg/mL. Gel permeation chromatography (GPC) analysis was performed with a Varian Prostar equipped with autosampler model 400, a PLgel 5 mm mixed-D column, a Prostar 355 RI detector, and THF as eluent at a flow rate of 1 mL min^−1^ (20 °C). Polystyrene standards purchased from Agilent technologies(Santa Clara, CA, USA) were used for calibration. Galaxie software was used to operate the instrument and Cirrus software for data processing. Thermogravimetric analysis (TGA) was performed on an SDT Q 600 instrument (TA Instruments, New Castle, DE, USA) with Advantage software. The samples in Al_2_O_3_ cups were heated from 30 to 600 °C with a ramp rate of 20 °C/min and a flow rate of 100 mL/min of N_2_ (furnace purge gas). Differential scanning calorimetry (DSC) measurements were obtained on a Perkin Elmer Jade (Waltham, MT, USA) differential scanning calorimeter and the instrument was calibrated using zinc and indium standards. Samples were prepared in aluminum pans. All polyesters were analyzed using the following heating program: −30 °C to 200 °C at 20 °C/min. The glass transition temperature (*T_g_*) of the polymer samples were determined from the second heating cycles with a heating/cooling rate of 10 °C/min under nitrogen atmosphere (20 mL/min). DSC data were analyzed using Pyris V9.0.2 software. The reported *T_g_* values are the average of three runs, and all of the individual runs were from the second heating cycles of fresh polyesters samples.

### 3.1. General Procedure for Copolymerization and Terpolymerization

A flame dried Schlenk flask was charged with a mixture of catalyst (0.5 mol%), maleic anhydride and epoxide(s) in toluene (4 mL) in a glovebox under nitrogen. The flask was maintained at the desired temperature using an oil bath controlled with a thermocouple. Small aliquots of the reaction mixture were collected at different time intervals to monitor the reaction with ^1^H NMR spectroscopy. The stirring was continued until nearly complete conversion of epoxide was observed, at which point the reaction was stopped and the mixture concentrated in vacuo. The resulting polymers were purified by dissolving the crude reaction mixture in methylenechloride (1–3 mL), followed by addition of methanol (4–5 mL), aided by cooling with liquid nitrogen. The solid polymer products were collected and dried in vacuo until constant weight was obtained. Purified polymers were characterized by various NMR and GPC techniques.

### 3.2. A Specific Example of Maleic Anhydride and Cyclohexene Oxide Using Cat-***1***

A flame dried Schlenk flask was loaded with a mixture of cat-**1** (10.0 mg, 0.0187 mmol, 0.5 mol%), maleic anhydride (3.75 mmol) and cyclohexene oxide (3.75 mmol) in toluene (4 mL) in a glovebox. The flask was heated to 100 °C and stirred for 2–9 h. The conversion of cyclohexene oxide was confirmed by ^1^H NMR spectroscopy and the reaction mixture was then concentrated in vacuo. The polymer was precipitated from addition of DCM (1–3 mL) and methanol (4–5 mL). After filtration, the polymer was dried in vacuo until constant weight was noted.

### 3.3. Examples of Terpolymerization using Cat-***1***

For the one-step terpolymerization, a flame dried Schlenk flask was loaded with a mixture of cat-**1** (10.0 mg, 0.0187 mmol, 0.5 mol%), maleic anhydride (7.5 mmol), cyclohexene oxide (3.75 mmol), styrene oxide (3.75 mmol) in toluene (4 mL) in a glovebox. The flask was heated to 100 °C and stirred for 2–9 h. After the complete conversion of epoxides was confirmed by ^1^H NMR spectroscopy, the reaction mixture were concentrated in vacuo. The polymer was precipitated from addition of DCM (1–3 mL) and methanol (4–5 mL). After filtration, the polymer was dried in vacuo until constant weight.

For the two-step terpolymerization using cat-**1**, a flame dried Schlenk flask was loaded with a mixture of cat-**1** (10.0 mg, 0.0187 mmol, 0.5 mol%), maleic anhydride (3.75 mmol) and cyclohexene oxide (3.75 mmol) in toluene (4 mL) in a glovebox. The flask was heated to 100 °C and stirred for 2–9 h, until the complete conversion of CHO was confirmed by ^1^H NMR spectroscopy. In the second step, to the same flask styrene oxide (3.75 mmol), and maleic anhydride (3.75 mmol) was added in a glovebox. The flask was again heated to 100 °C and stirred for 0.5–1 h. The conversion of SO was confirmed by ^1^H NMR spectroscopy and the reaction mixture were concentrated in vacuo. The polymer was precipitated from addition of DCM (1–3 mL) and methanol (4–5 mL). After filtration, the polymer was dried in vacuo until constant weight.

## 4. Conclusions

In summary, we have demonstrated that the amido-oxazolinate zinc complexes 1–4 are effective catalysts for the ROCOP of maleic anhydride with various epoxides, leading to the synthesis of unsaturated polyesters with easily modifiable backbones. The relative activity of epoxides (SO > CHO > PGE) in these reactions can be correlated with the electronic and steric features of the substrates, along with considerations of the chain propagation steps. The reactivity of the zinc catalysts is further exploited to prepare terpolymers from one anhydride and two epoxides, providing additional structural variability for the polyesters. Future studies will focus on the development of new catalysts as well as synthesis of copolymers with controlled segments and their potential applications.

## Figures and Tables

**Table 1:** Optimization of reaction conditions between maleic anhydride (MA) and cyclohexene oxide (CHO).^[a].^

Entry	[cat]:[CHO]:[MA]	Time	Convn ^[b]^	Ester (%) ^[b]^	*M*_n_^^[c]^^ (kg/mol)	Đ ^^[c]^^
1	0:100:100	24	0	-	-	-
2	1:100:100	16	100	53	5.9	1.29
3 ^[d]^	1:100:100	24	100	73	2.7	1.43
4	1:200:200	9	100	61	2.3	1.20
5 ^[e]^	1:200:200	16	96	65	1.2	1.19
6 ^[f]^	1:200:200	0.92	88	14	3.8	1.82
7	1:200:400	1	100	46	0.78	1.12
8	1:1000:1000	1.5	100	34	1.2	1.28

^[a]^ All reactions were performed using cat-**1** in toluene (2 mL) at 100 °C. ^[b]^ The conversion of CHO and the ester% were determined by measuring the intensities of peaks by ^1^H NMR spectroscopy. ^[c]^ Determined by gel permeation chromatography (GPC) using tetrahydrofuran (THF) as solvent. ^[d]^ CHO in toluene was added slowly to a solution of catalyst and maleic anhydride. ^[e]^ 10 mL of toluene was used. ^[f]^ Bulk reaction without toluene.

**Table 2:** ROCOP of MA with different Epoxides ^[a]^.

Entry	Epoxides	Time (h)	Convn (%)	Ester (%)	*M*_n_ (kg mol^−1^)	Đ
1	SO	0.33	100	76	2.6	1.13
2	CHO	9	100	61	2.3	1.20
3	PGE	48	100	95	4.8	2.05

^[a]^ All reactions were performed using cat-**1** in toluene (2 mL) at 100 °C with [cat]:[MA]:[epoxide] = 1:200:200. The conversion of epoxides and the ester % were determined by ^1^H NMR spectroscopy.

**Table 3:** ROCOP of CHO with different cyclic anhydrides ^[a]^.

Entry	Cat	Anhydride	Time (h)	Convn (%) ^[b]^	Ester (%) ^[b]^	*M*_n_^^[c]^^ (kg mol^−1^)	Đ ^^[c]^^
1	**1**	MA	9	100	61	2.3	1.20
2	**2**	MA	3.5	100	44	2.6	1.30
3	**3**	MA	2	100	37	5.3	1.30
4	**4**	MA	2	100	40	2.9	1.34
5	**1**	PA	3	93	37	1.5	1.17
6	**2**	PA	5.5	97	40	1.8	1.24
7	**3**	PA	3	100	41	1.9	1.15
8	**4**	PA	4.5	97	44	1.8	1.20
9	**1**	SA	9	98	60	3.7	1.24
10	**2**	SA	5.5	78	74	4.2	1.30
11	**3**	SA	9	91	55	4.2	1.15
12	**4**	SA	11	98	67	3.3	1.14

^[a]^ All reactions were performed with [CHO]:[anhydride]:[cat] = 200:200:1 in toluene (2 mL) at 100 °C. ^[b]^ The conversion of CHO and the ester % were determined by ^1^H NMR spectroscopy. ^[c]^ Determined by GPC using THF as solvent.

**Table 4:** Terpolymerization of MA with two different epoxides ^[a].^

Run	Step 1	Step 2	Ester Ratio ^[b]^	*M*_n_^^[c]^^ (kg mol^−1^)	Đ ^^[c]^^
1	CHO/MA	PGE/MA	CHO:PGE = 1.0:2.3	4.8	2.21
2	PGE/MA	CHO/MA	CHO:PGE = 1.0:2.7	10.3	1.88
3	SO/MA	CHO/MA	CHO:SO = 1.0:3.8	3.9	1.41
4	CHO/PGE/MA		CHO:PGE = 1.0:1.7	4.3	1.88
5	CHO/SO/MA		SO:CHO = 1.0:0.94	2.0	1.34
6	SO/PGE/MA		SO:PGE = 1.0:1.0	1.9	1.40

^[a]^ All reactions were performed using cat-**1** in toluene (2 mL) at 100 °C. The first three were run in two steps while the last three were run in a single step with both epoxides present from the start. ^[b]^ The ester ratios were determined from the intensities of the corresponding ester peaks in the polymers by ^1^H NMR spectroscopy. ^[c]^ Determined by GPC using THF as eluent.

**Table 5:** Thermal properties of co- and terpolymers ^[a].^

Entry	Polymer	*T_g_*	*T*_−5%_	*T*_−50%_
Table 2, entry 1	p(SO-MA)	39	256	314
Table 2, entry 2	p(CHO-MA)	63	211	349
Table 2, entry 3	p(PGE-MA)	30	244	387
Table 4, entry 1	p(CHO-PGE-MA)	41	213	375
Table 4, entry 2	p(CHO-PGE-MA)	44	281	370
Table 4, entry 3	p(SO-CHO-MA)	61	219	352
Table 4, entry 4	p(CHO-PGE-MA)	43	208	366
Table 4, entry 5	p(CHO-SO-MA)	59	156	316
Table 4, entry 6	p(SO-PGE-MA)	24	199	350

^[a]^ Temperatures in °C. *T_g_* values were determined from the second heating cycle in differential scanning calorimetry (DSC). *T*_−5%_ and *T*_−50%_, refer to the temperatures at which 5% and 50% weight losses were observed in thermogravimetric analysis (TGA), respectively.

## Supplementary Materials

The following are available online: Figures S1–S18: 1H and 13C NMR spectra of selected polyester samples from ROCOP; Figure S19, DTA plot of a one-step terpolymer p(CHO-SO-MA).
