# Supplementary material for: Zinc Amido-Oxazolinate Catalyzed Ring Opening Copolymerization and Terpolymerization of Maleic Anhydride and Epoxides
Source: Molecules. 2020 Sep 4;25(18):4044. doi: 10.3390/molecules25184044 (PMC7570669; doi:10.3390/molecules25184044)
Supplement: Supplementary file 1 [file molecules-25-04044-s001.pdf]

Zinc Amido-Oxazolinate Catalyzed Ring Opening Copolymerization and Terpolymerization of Maleic Anhydride and Epoxides

Muneer Shaik, Vamshi K. Chidara, Srinivas Abbina, Guodong Du

Department of Chemistry, University of North Dakota, 151 Cornell Street Stop 9024, Grand Forks, North Dakota 58202, United States.

Email: guodong.du@und.edu

Table of Contents

Figure S1. ^{1}H NMR spectrum of poly(CHO-MA) from ROCOP of CHO & MA with catalyst **1** 3

Figure S2. ^{13}C NMR spectrum of poly(CHO-MA) from ROCOP of CHO & MA with catalyst **1** 3

Figure S3. ^{1}H NMR spectrum of poly(CHO-SA) from ROCOP of CHO & SA with catalyst **1** 4

Figure S4. ^{1}H NMR spectrum of poly(CHO-PA) from ROCOP of CHO & PA with catalyst **1** 4

Figure S5. ^{1}H–^{13}C HETCOR spectrum of poly(PGE-MA) from ROCOP of PGE, & MA with catalyst **1** 5

Figure S6. ^{1}H-^{1}H COSY-NMR spectrum of poly(PGE-MA) from ROCOP of PGE, & MA with catalyst **1** 5

Figure S7. ^{1}H NMR spectrum of poly(PGE-SO-MA) from ROCOP of PGE, SO& MA with catalyst **1** Table 4, entry 6 6

Figure S8. ^{13}C NMR spectrum of poly(PGE-SO-MA) from ROCOP of PGE, SO & MA with catalyst **1** 6

Figure S9. ^{1}H NMR spectrum of poly(PGE-CHO-MA) from ROCOP of PGE, CHO & MA with cat-**1** Table 4, entry 4 7

Figure S10. ^{13}C NMR spectrum of poly(PGE-CHO-MA) from ROCOP of PGE, CHO&MA with cat-**1** 7

Figure S11. ^{1}H NMR spectrum of poly(SO-CHO-MA) from ROCOP of CHO, SO & MA with catalyst **1** (Table 4, entry 5)........ 8

Figure S12. ^{13}C NMR spectrum of poly(SO-CHO-MA) from ROCOP of CHO, SO & MA with catalyst **1** 8

Figure S13. ^{1}H NMR spectrum of poly(CHO-PGE-MA) from ROCOP of CHO, PGE & MA 9

Figure S14. ^{13}C NMR spectrum of poly(CHO-PGE-MA) from ROCOP of CHO, PGE & MA 9

Figure S15. ^{1}H NMR spectrum of poly(PGE-CHO-MA) from ROCOP of PGE, CHO & MA (Two step addition, Table 4, entry 2)........ 10

Figure S16. ^{13}C NMR spectrum of poly(PGE-CHO-MA) from ROCOP of PGE, CHO & MA (Two step addition, Table 4, entry 2) 10

Figure S17. ^{1}H NMR spectrum of poly(SO-CHO-MA) from ROCOP of CHO, SO & MA with catalyst **1** (Two step reaction Table 4, entry 3) 11

Figure S18. ^{13}C NMR spectrum of poly(SO-CHO-MA) from ROCOP of CHO, SO & MA with catalyst **1** (Two step reaction Table 4, entry 3)........ 11

Figure S19. DTA plot of a one-step terpolymer p(CHO-SO-MA) (Table 4, entry 5) 12

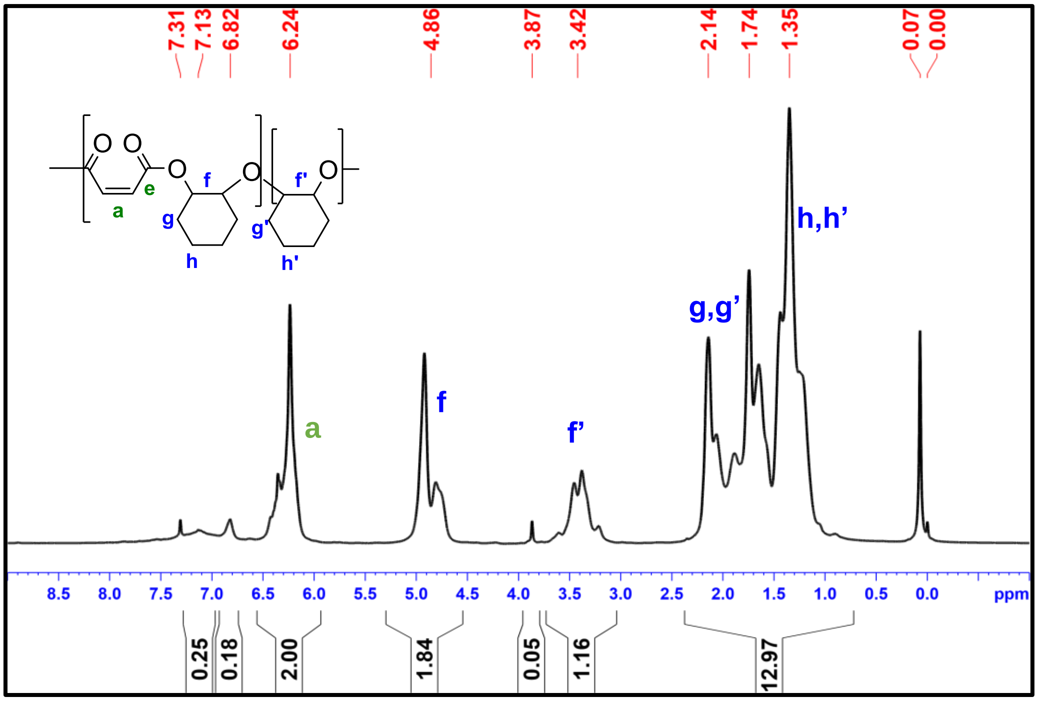

Figure S1. ^{1}H NMR spectrum of poly(CHO-MA) from ROCOP of CHO & MA with catalyst **1**

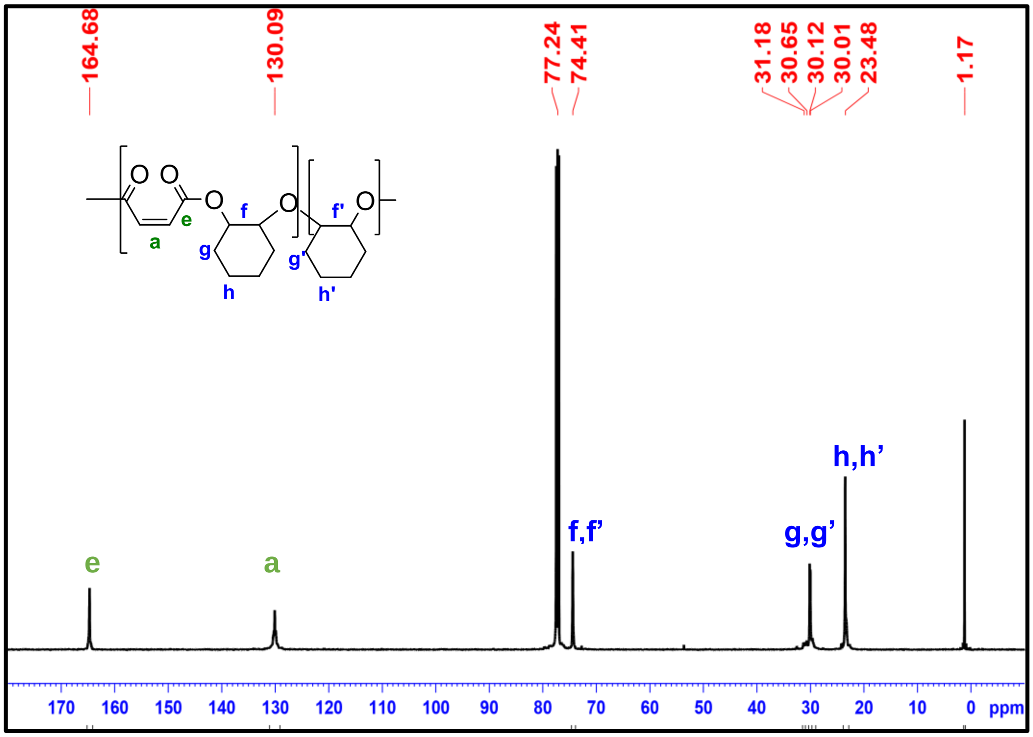

Figure S2. ^{13}C NMR spectrum of poly(CHO-MA) from ROCOP of CHO & MA with catalyst **1**

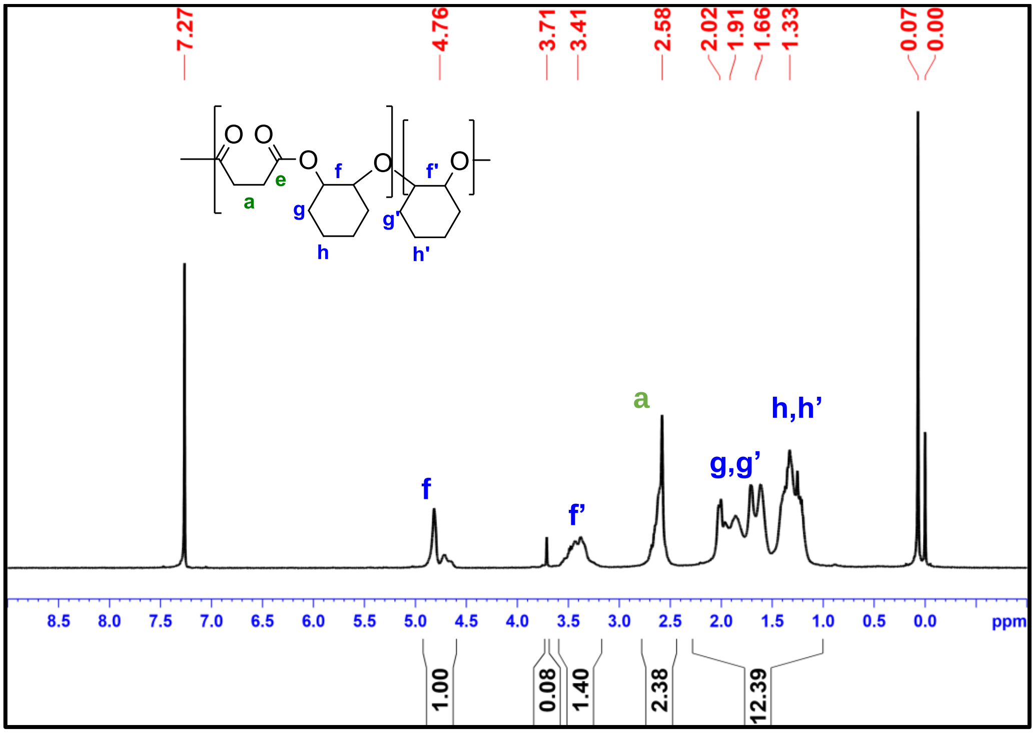

Figure S3. ^{1}H NMR spectrum of poly(CHO-SA) from ROCOP of CHO & SA with catalyst **1**

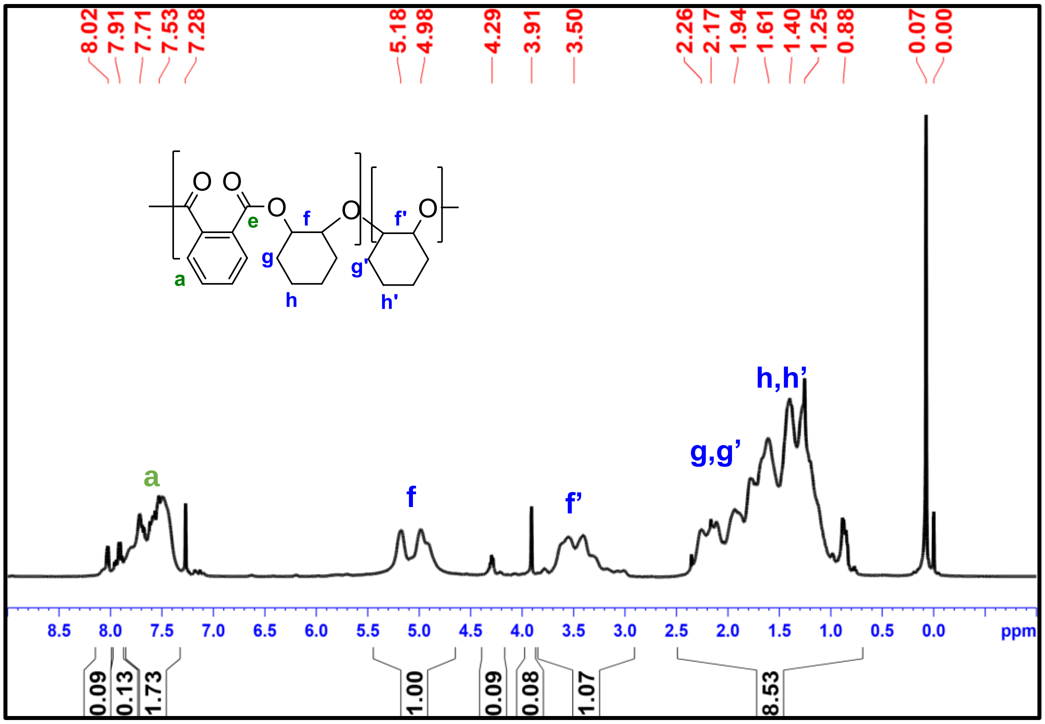

Figure S4. ^{1}H NMR spectrum of poly(CHO-PA) from ROCOP of CHO & PA with catalyst **1**

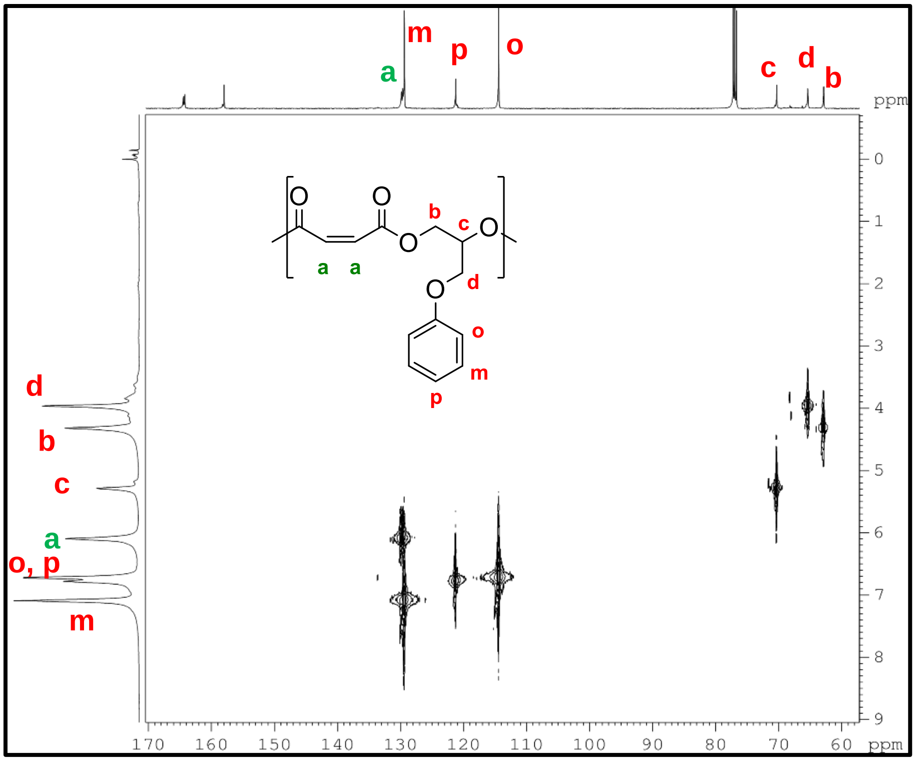

Figure S5. ^{1}H–^{13}C HETCOR spectrum of poly(PGE-MA) from ROCOP of PGE, & MA with catalyst **1**

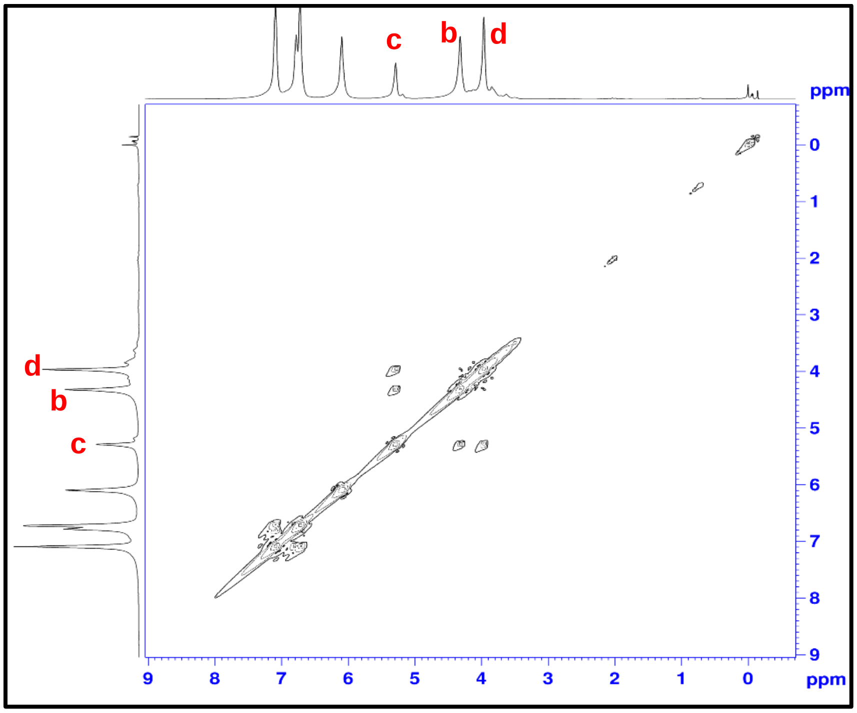

Figure S6. ^{1}H-^{1}H COSY-NMR spectrum of poly(PGE-MA) from ROCOP of PGE, & MA with catalyst **1**

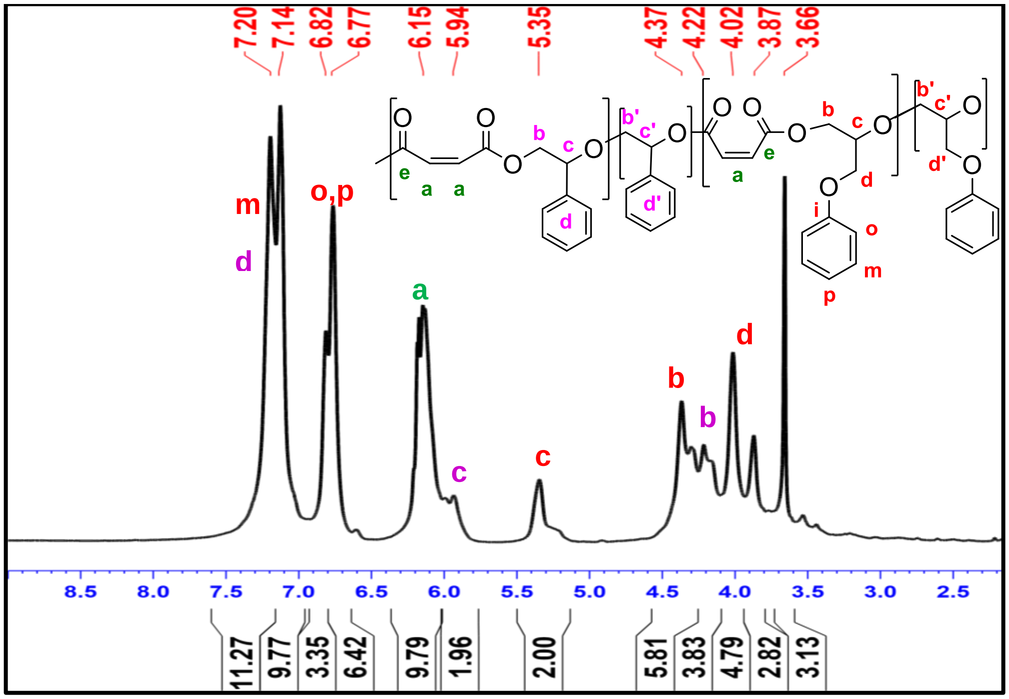

Figure S7. ^{1}H NMR spectrum of poly(PGE-SO-MA) from ROCOP of PGE, SO& MA with catalyst **1** Table 4, entry 6

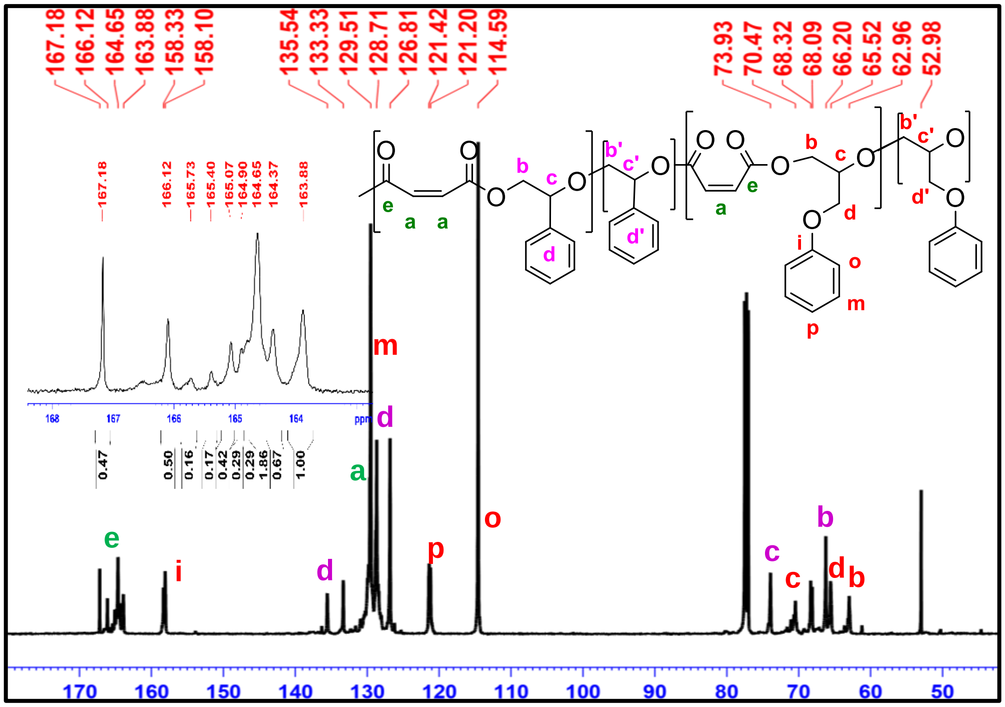

Figure S8. ^{13}C NMR spectrum of poly(PGE-SO-MA) from ROCOP of PGE, SO & MA with catalyst **1**

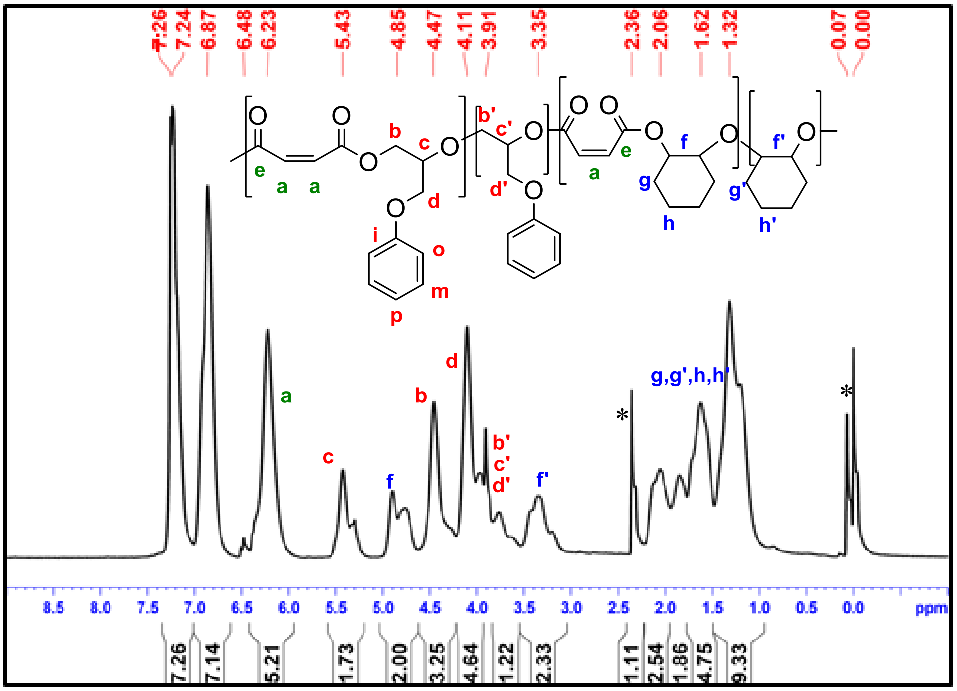

Figure S9. ^{1}H NMR spectrum of poly(PGE-CHO-MA) from ROCOP of PGE, CHO & MA with cat-**1** Table 4, entry 4

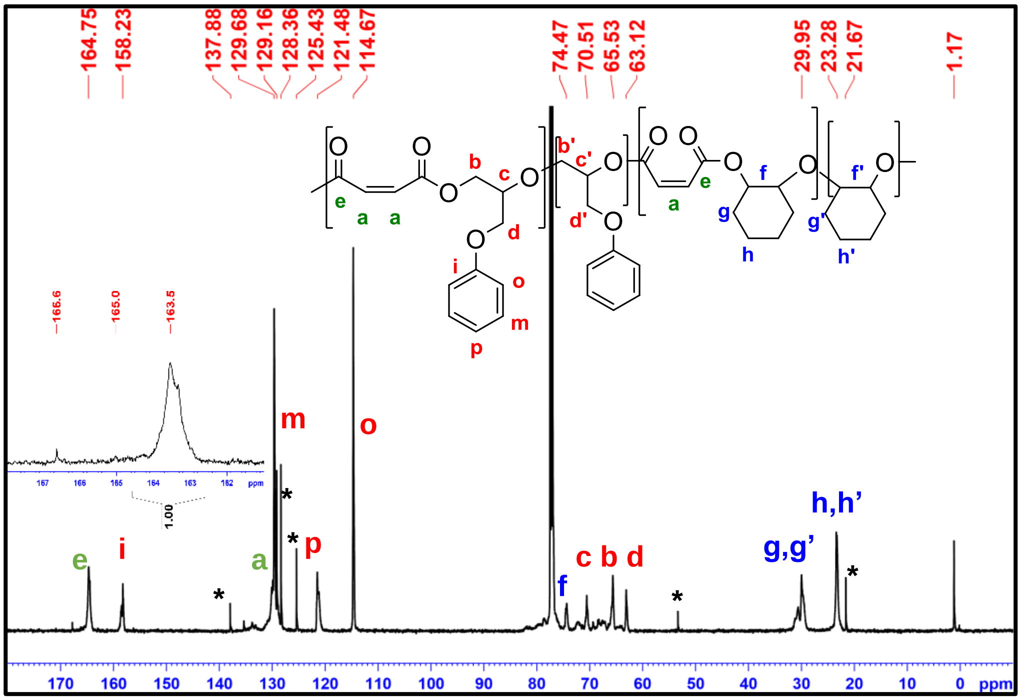

Figure S10. ^{13}C NMR spectrum of poly(PGE-CHO-MA) from ROCOP of PGE, CHO&MA with cat-**1**

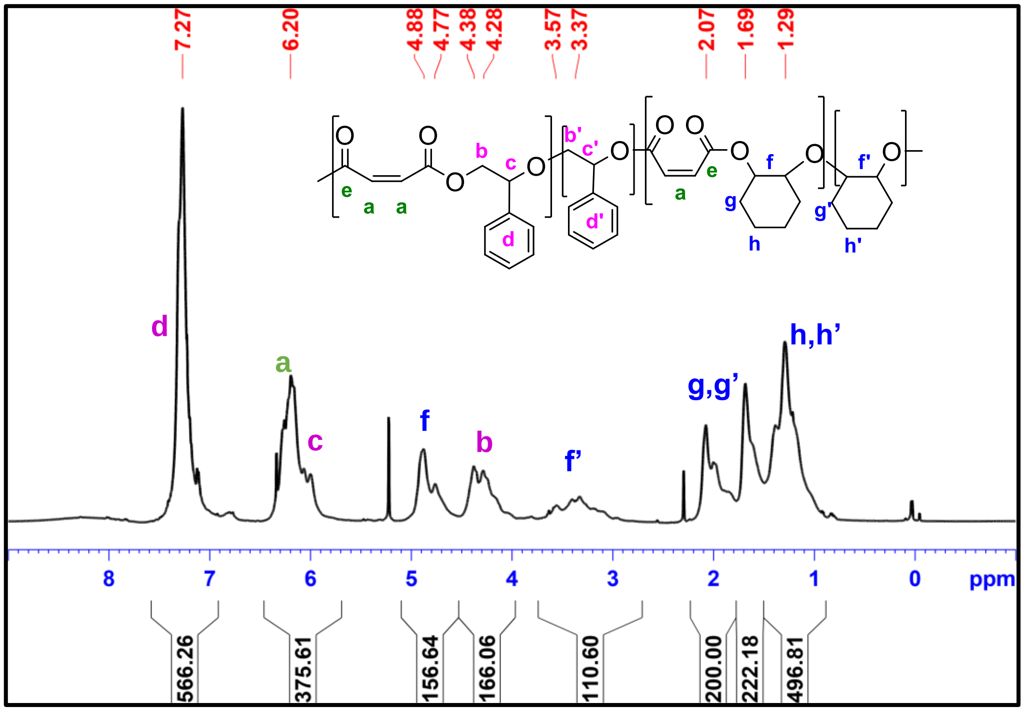

Figure S11. ^{1}H NMR spectrum of poly(SO-CHO-MA) from ROCOP of CHO, SO & MA with catalyst **1** (Table 4, entry 5)

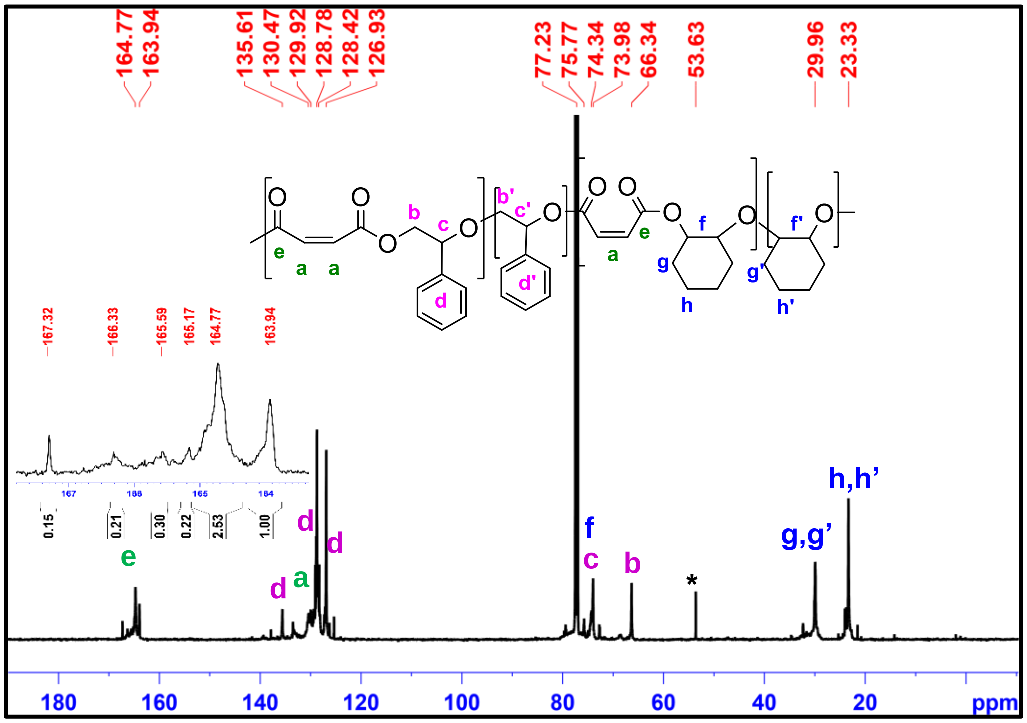

Figure S12. ^{13}C NMR spectrum of poly(SO-CHO-MA) from ROCOP of CHO, SO & MA with catalyst **1**

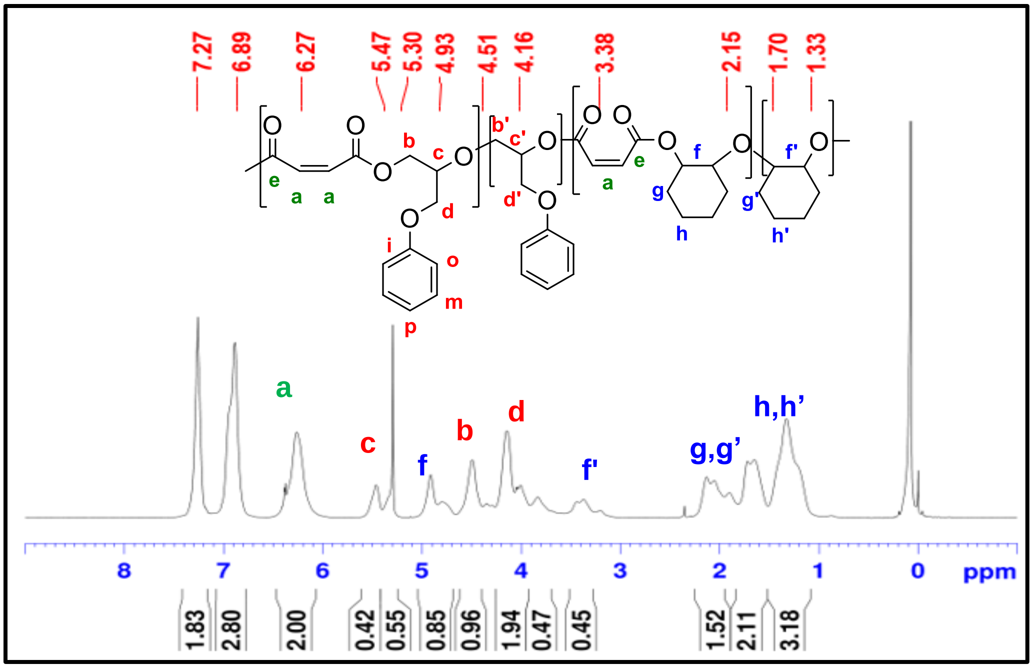

Figure S13. ^{1}H NMR spectrum of poly(CHO-PGE-MA) from ROCOP of CHO, PGE & MA (Two step addition, Table 4, entry 1)

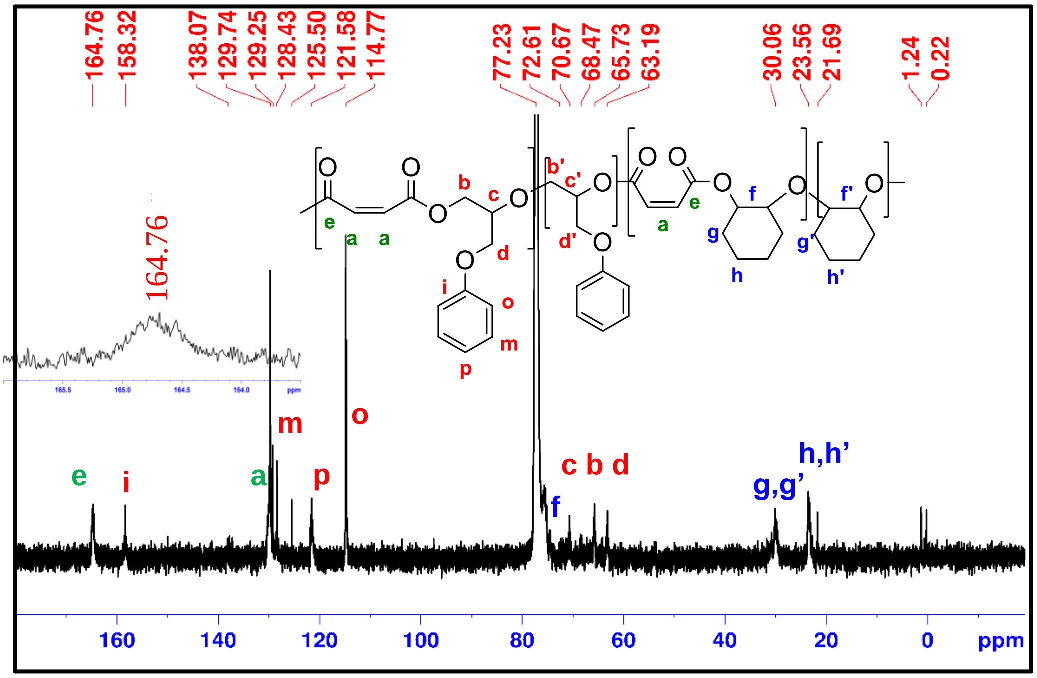

Figure S14. ^{13}C NMR spectrum of poly(CHO-PGE-MA) from ROCOP of CHO, PGE & MA (Two step addition)

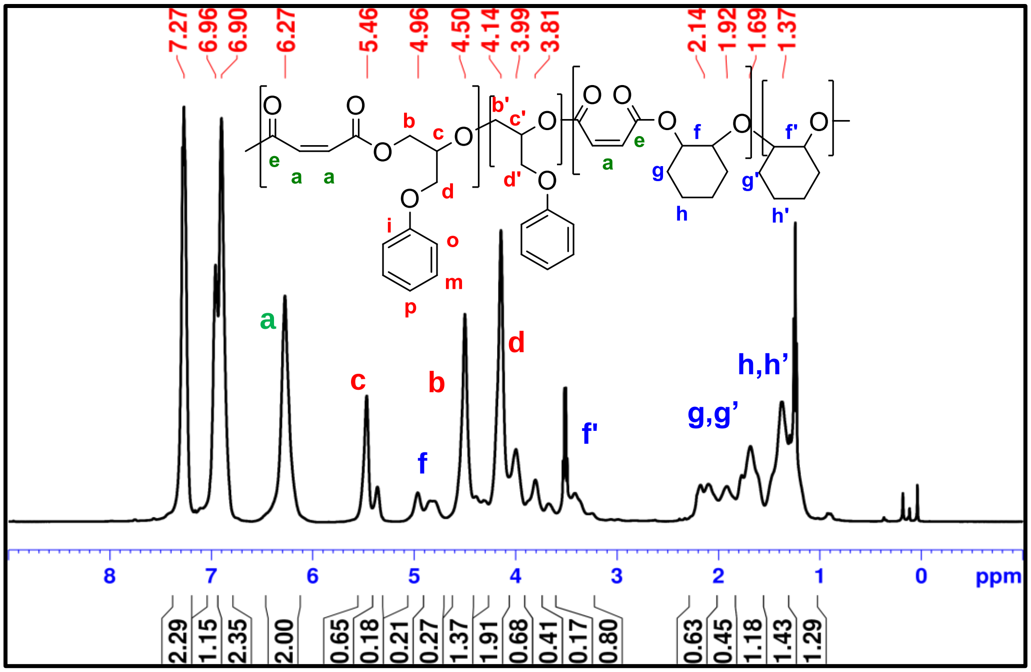

Figure S15. ^{1}H NMR spectrum of poly(PGE-CHO-MA) from ROCOP of PGE, CHO & MA (Two step addition, Table 4, entry 2)

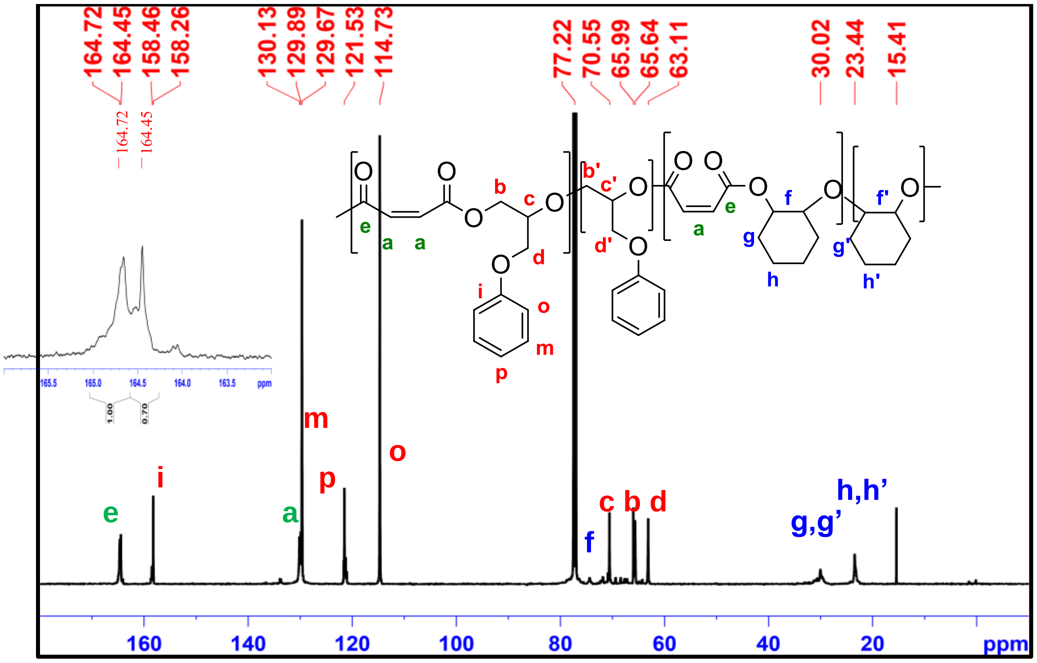

Figure S16. ^{13}C NMR spectrum of poly(PGE-CHO-MA) from ROCOP of PGE, CHO & MA (Two step addition, Table 4, entry 2)

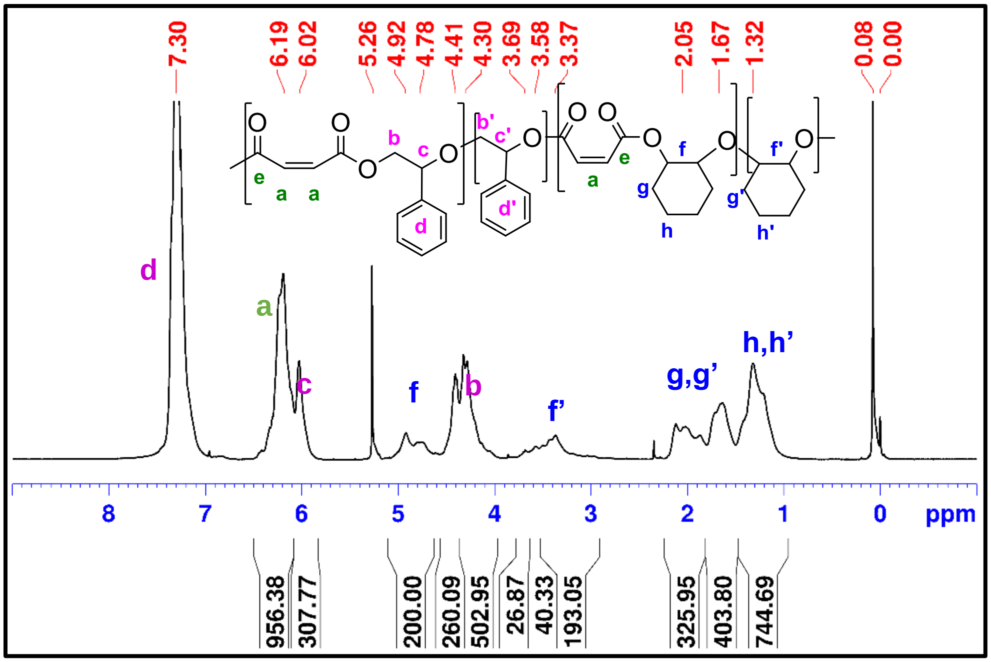

Figure S17. ^{1}H NMR spectrum of poly(SO-CHO-MA) from ROCOP of CHO, SO & MA with catalyst **1** (Two step reaction Table 4, entry 3)

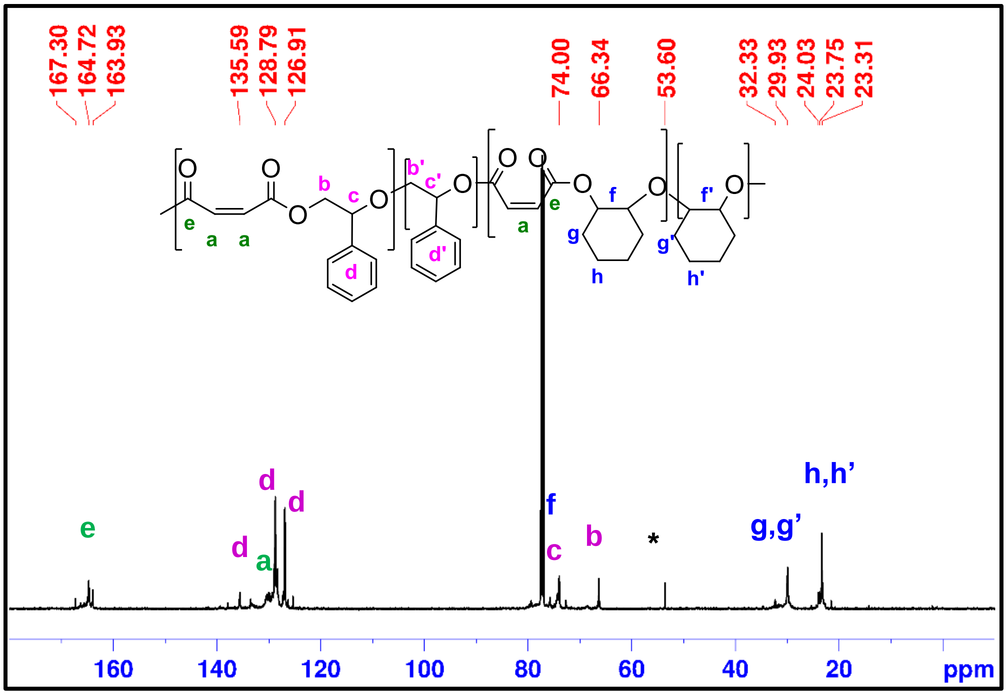

Figure S18. ^{13}C NMR spectrum of poly(SO-CHO-MA) from ROCOP of CHO, SO & MA with catalyst **1** (Two step reaction Table 4, entry 3)

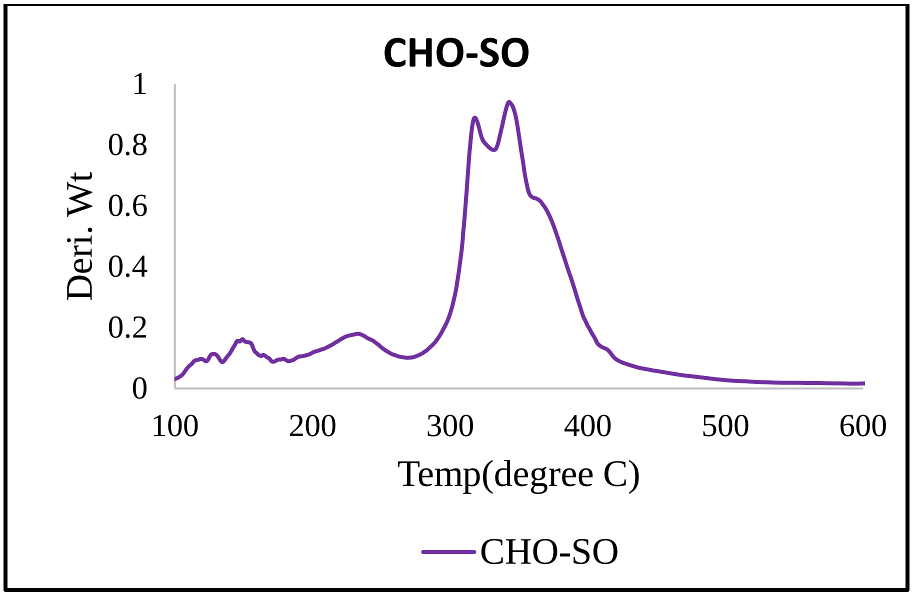

Figure S**19**. DTA plot of a one-step terpolymer p(CHO-SO-MA) (Table 4, entry 5)
